# Deep Learning Model to Predict Serious Infection Among Children With Central Venous Lines

**DOI:** 10.3389/fped.2021.726870

**Published:** 2021-09-15

**Authors:** Azade Tabaie, Evan W. Orenstein, Shamim Nemati, Rajit K. Basu, Gari D. Clifford, Rishikesan Kamaleswaran

**Affiliations:** ^1^Department of Biomedical Informatics, Emory School of Medicine, Atlanta, GA, United States; ^2^Department of Pediatrics, Emory University School of Medicine, Atlanta, GA, United States; ^3^Department of Biomedical Informatics, University of California, San Diego, San Diego, CA, United States; ^4^Department of Biomedical Engineering, Georgia Institute of Technology and Emory School of Medicine, Atlanta, GA, United States

**Keywords:** explainable machine learning, infection, PSI, predictive model, sepsis

## Abstract

**Objective:** Predict the onset of presumed serious infection, defined as a positive blood culture drawn and new antibiotic course of at least 4 days (PSI^*^), among pediatric patients with Central Venous Lines (CVLs).

**Design:** Retrospective cohort study.

**Setting:** Single academic children's hospital.

**Patients:** All hospital encounters from January 2013 to December 2018, excluding the ones without a CVL or with a length-of-stay shorter than 24 h.

**Measurements and Main Results:** Clinical features including demographics, laboratory results, vital signs, characteristics of the CVLs and medications used were extracted retrospectively from electronic medical records. Data were aggregated across all hospitals within a single pediatric health system and used to train a deep learning model to predict the occurrence of PSI^*^ during the next 48 h of hospitalization. The proposed model prediction was compared to prediction of PSI^*^ by a marker of illness severity (PELOD-2). The baseline prevalence of line infections was 0.34% over all segmented 48-h time windows. Events were identified among cases using onset time. All data from admission till the onset was used for cases and among controls we used all data from admission till discharge. The benchmarks were aggregated over all 48 h time windows [N=748,380 associated with 27,137 patient encounters]. The model achieved an area under the receiver operating characteristic curve of 0.993 (95% CI = [0.990, 0.996]), the enriched positive predictive value (PPV) was 23 times greater than the base prevalence. Conversely, prediction by PELOD-2 achieved a lower PPV of 1.5% [0.9%, 2.1%] which was 5 times the baseline prevalence.

**Conclusion:** A deep learning model that employs common clinical features in the electronic health record can help predict the onset of CLABSI in hospitalized children with central venous line 48 hours prior to the time of specimen collection.

## Introduction

Central line-associated bloodstream infections (CLABSIs) are a major cause of healthcare-associated infections among hospitalized children and contribute to increased morbidity, length of hospital stay, and cost (1, 2). The U.S. Centers for Disease Control and Prevention (CDC) estimates that approximately 80,000 new CLABSIs occur in the United States every year, and data show a 12–25% increased risk of mortality in hospitalized patients who develop a CLABSI (3, 4). Early identification of the onset of infections such as CLABSI or sepsis can prevent adverse outcomes, reduce costs, and improve the quality of care (5, 6).

While specific definitions for entities such as CLABSI and sepsis exist in pediatrics, they often have inadequate sensitivity for clinically important infections and may be difficult to generalize across electronic medical record (EMR) platforms (7, 8). Presumed serious infection (PSI), which is used in both adult and pediatric sepsis surveillance systems, is defined as a blood culture being obtained (regardless of the result) followed by new antimicrobial agents started within 2 days of the blood culture (i.e., agents that were not being administered prior to the blood culture) that are administered for at least 4 consecutive days or until time of death or transfer to another hospital (9–11). This PSI definition captures *suspicion* for infection (as identified by obtaining a blood culture) along with sufficient antimicrobial use to distinguish empirical treatment of a suspected infection from definitive treatment. Successful prediction of PSI, or sepsis in general, among hospitalized children or the adult population could expedite recognition and initiation of therapy (5).

Machine learning models have the potential to predict the onset of infection prior to clinical suspicion, allowing clinicians to take preventive measures and reduce mortality and morbidity (12–15). However, one of the main challenges in employing machine learning models in the clinical domain is that many events worthy of prediction are uncommon, also known as the extremely class-imbalanced dataset problem (16). For example, in the pediatric cardiac intensive care unit (ICU), Alten et al. found that hospital acquired infection occurred in 2.4% of CICU encounters at a rate of 3.3/1000 CICU days (17). To date, studies to predict CLABSI onset have mainly investigated known clinical risk factors associated with the infection and developed discriminative models based on non-temporal data (18, 19). While these approaches may be able to predict if a CLABSI will occur during an entire hospital visit or not, their performance likely decreases when considering the next 48–72 h of a patient's care. Real-time predictions that estimate the risk of an adverse event in a defined time window are more useful clinically, but they are more challenging to develop because the prevalence of the event in a defined time window is lower than its prevalence across an entire hospital stay (20, 21). Currently a CLABSI prediction tool does not exist and instead providers use either subjective information or derived metrics such as severity of illness scores. In pediatrics, a commonly used severity of illness score is the PEdiatric Logistic Organ Dysfunction (PELOD) score. PELOD has been used to predict death and need or duration of intensive care unit resources (22).

Most traditional machine learning algorithms assume a balanced distribution of negative and positive samples in the data (i.e., a prevalence close to 50%). Deep learning models have the potential to overcome these limitations as they are more capable of finding patterns in extremely class-imbalanced high-dimensional data. However, deep learning models are commonly thought of as impossible to understand, overly complex, and not pragmatic. These models' lack of explainability may reduce their implementation effectiveness even with good predictive performance.

In this study, we aimed to develop a pragmatic deep learning framework that can adequately predict the onset of presumed bloodstream infection in children with a central line during the next 48 h of their hospitalization. At each point of prediction, the model provides insights to its decision-making process by outputting the effect of the most influential features on the predicted outcome.

## Materials and Methods

### Study Design

A retrospective cohort study was conducted which included all hospitalized patients with a central venous line (CVL) at a single tertiary care pediatric health system. The inclusion criteria for patients were (1) admission to one of three freestanding children's hospitals between January 1st, 2013 and December 31st, 2018, (2) having a documented CVL at some point during the hospitalization (e.g., present and not yet removed at the time of admission or placed during the hospitalization), and (3) having length-of-stay longer than 24 h. As described earlier, our goal is not to identify causes of presumed bloodstream infection associated with CVL, but rather predict the infection among patients with CVL. The predictive model was developed as it would be applied in clinical practice; therefore, we included both patients whose line was placed within the local health system or before admission. If CVL was placed within the local health system, information about line placement, such as sterile technique, was included. For patients whose line was not placed within the local health system, those data were not available to the model, just as they would not be available in the EHR when making a prediction in real clinical practice. This study was conducted according to Emory University protocol number 19-012.

### Outcome Definition

We defined our primary outcome as a presumed serious infection (PSI) along with a laboratory confirmed bloodstream infection defined as a positive blood culture (9, 10). We reviewed this definition through informal interviews with 2 pediatric infectious disease specialists, 1 pediatric critical care physician, 1 neonatologist, and 1 pediatric hematology/oncology specialist to validate its appropriateness and clinical utility. From this point, we referred to *PSI with positive blood culture* as PSI^*^ for clarity reasons.

### Feature Extraction

The extracted features from the EHR were demographics, laboratory results, vital signs, prior diagnoses, microbiology results, medications, respiratory support, CVL information, and CVL care documentation. We focused on features anticipated to be routinely recorded in the EHR across centers. The full list of extracted features and the preprocessing steps are available in Appendix A.

As initial deep learning techniques are often exploratory, it is true that many variables would on the surface seem unrelated. While biopathophysiologic links can indeed be created related to escalating PEEP (e.g., worsening microvascular/endothelial injury in the pulmonary vasculature potentially related to cytokine storm/inflammation as a response to a brewing infection or pulmonary edema from endovascular injury and leak and fluid delivery) – the beauty of a deep learning model approach is it reduces clinician bias that a variable (or set of variables) is or is not related to the outcome of interest. As the literature shows – many models have been able to identify constellations of variables that would go otherwise unheeded as heralds to a patient event (11, 19).

### Window-Wise Study Design

The onset of PSI^*^ is defined as a positive blood culture time after a CVL was inserted, succeeded by a new antibiotic administration for at least four days. Hospitalized patients in the cohort could have a CVL at the time of admission or received at least one during hospitalization time. We restricted our analysis to blood cultures with specimen collection timestamps while the patient had a CVL during hospitalization. A patient may become infected multiple times during a single hospitalization. However, for the purposes of this analysis, we censored hospitalizations after the first PSI^*^ event for a patient if present.

**Figure 1:**
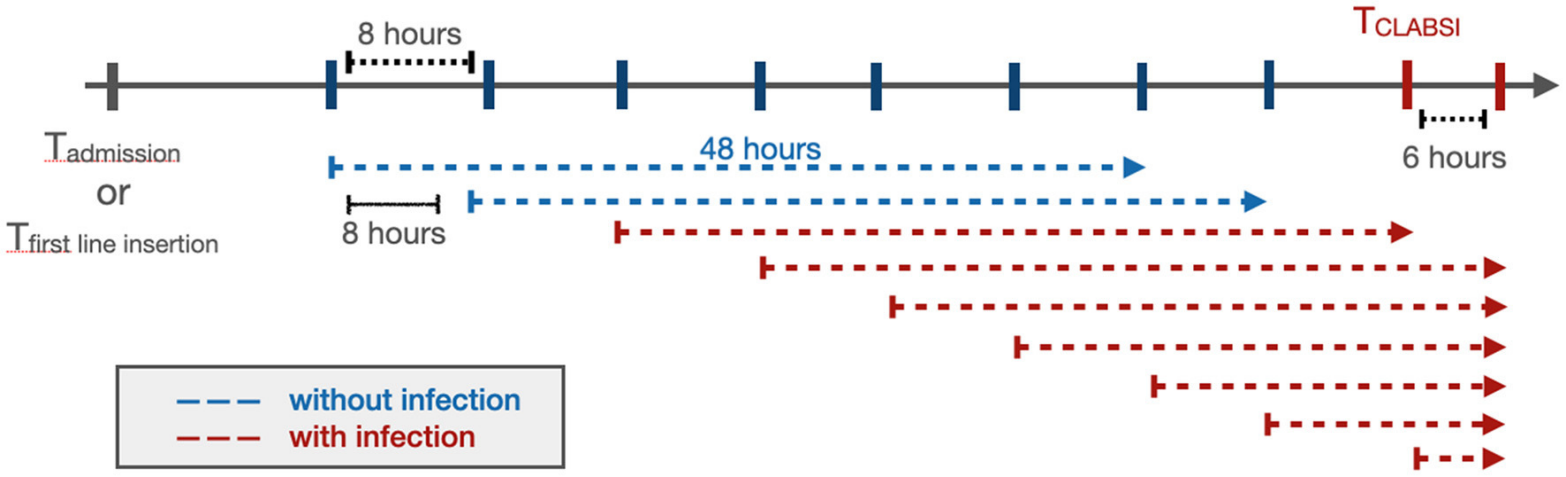
The window-wise study design. If a patient had a documented CVL that was not documented as removed at the time of admission, the start point of the analysis would be the admission time. Otherwise, the start point would be the first line insertion time. The prediction window was 48 h with an 8 h sliding window until the end of the patient's hospitalization or removal of the last CVL. When the onset of CLABSI occurred within a 48 h prediction window, that window was considered positive (red), while the rest (blue) were labeled as negative. The prediction was performed at the start of each arrow.

To predict the onset of PSI^*^ in a real-time setting, we used a window-wise study design (Figure 1). We started monitoring a patient from admission or first line insertion time, whichever was earlier. We then aimed to predict whether a patient would have a PS^*^ in the next window of 48 h; this prediction window was selected to give health providers enough time to intervene to potentially prevent a PSI^*^, for example by removing high risk CVLs or other interventions. Every 8 h, the model would incorporate new information obtained and make another prediction for the subsequent 48 h. The 8 h sliding window was selected to reflect the cadence of shift changes and rounds, particularly in the ICUs at our institution. Even if the windows do not correspond specifically to shift changes and rounds, we nonetheless felt that more frequent updates would yield more relevant information for clinicians. All 48 h windows that included a PSI^*^ time were labeled as positive and the rest were negative.

The patient encounters were split into training (80%) and testing (20%). The train-test split procedure is used to estimate the performance of machine learning algorithms when they are used to make predictions on data not used to train the model. We followed the commonly used 80-20 split in order to provide enough examples for the models to learn. Additionally, 10% of the training set was used as the validation set to optimize the model's settings and tune the model's hyperparameters. After preprocessing the data and removing collinearity, there were 135 features to feed into the prediction model. The list of 135 features and the details on preprocessing are described in Appendix A. The PSI^*^ prevalence in the window-wise study was 0.34%, meaning that approximately 1/300 of the 48 h time windows contained the onset of a PSI^*^.

### Models

Real-time prediction of PSI^*^ is an extremely class-imbalanced problem (see below). To tackle this challenge, we started with a Long Short-Term Memory (LSTM) model (23, 24), a recurrent neural network model capable of dealing with long sequences of data that has performed well for adult sepsis prediction (25). To improve the performance of this model on an extremely class-imbalanced dataset, we hypothesized that:

*Hypothesis 1: Penalizing false positives and false negatives in the optimization function (focal loss) will improve model performance*. In extremely class-imbalanced modeling, the model is biased towards the majority class which in our case is not having an onset of PSI^*^. In machine learning models, a loss function value is a measure of how far off a model's prediction is from the actual outcome value, and the algorithms are optimized to minimize this value. Focal loss reduces the loss of well-classified examples, emphasizing the false positives and negatives (26). We hypothesized that a focal loss function would improve performance relative to traditional methods for dealing with imbalanced data such as under-sampling the majority class.
*Hypothesis 2: Incorporating an attention mechanism will improve model performance*. An attention mechanism in deep learning assigns attention weights to source data at each time point, allowing the model to focus only on information relevant to the next prediction (27).

To evaluate these hypotheses, we developed and evaluated the following machine learning models: (1) a simple Bidirectional LSTM with binary cross-entropy, (2) a simple Bidirectional LSTM that was trained with an under-sampled majority class to make the labels more balanced, (3) a Bidirectional LSTM with Focal loss, and (4) a Bidirectional LSTM with Focal loss and an attention mechanism. More details on the proposed model are presented in Appendix B.

### Performance Metrics

For each model, we calculated the Area Under the Receiver Operating Characteristics Curve (AUROC), sensitivity, specificity and accuracy. We also calculated metrics that are more informative in extremely class-imbalanced data classification models such as Area Under Precision-Recall Curve (AUPRC), positive predictive value (PPV), negative predictive value (NPV) and F-1 score. The 95% confidence interval estimation for each metric was calculated using bootstrapping.

### Model Explainability

Decision making process of a deep learning model is often assumed to be overly complex. However, there are several ways to illuminate the decisions a model makes. It is also achievable to understand which features are the most salient in a model's prediction.

We estimated feature importance for each prediction by employing Shaply Additive exPlanations (SHAP) values, a method for explaining predictive models based on game theory (28). SHAP values presents the contribution of each feature to the model's decision-making process and their effect size on the predicted outcome. These SHAP values can be summarized across the cohort or calculated for an individual model prediction to inform clinicians of the features influencing a specific prediction, providing model transparency and observability to the end user (29).

### Clinical Benchmark

To make the model relevant, we compared performance against an existing model used for prediction of illness in hospitalized children. In the absence of a discrete prediction model used for prediction of line or bloodstream infections, we used the PEdiatric Logistic Organ Dysfunction 2 (PELOD-2) score. The PELOD-2 score has been validated for prediction of morbidity and mortality in hospitalized children. We calculated PELOD-2 at every prediction point, then considered different cut-off values to identify the PSI^*^ positive windows (28). Applying the same threshold values on the testing set, we predicted the PSI^*^ positive windows by the use of the corresponding PELOD-2 values for each prediction window.

Pediatric Risk of Mortality III (PRISM-III) has also been validated for mortality prediction in hospitalized children (30). Calculating PRISM-III score enables the physicians to identify which patients require more urgent care and interventions. We investigated the differences in PRISM-III components across PSI^*^ and non-PSI^*^ time windows.

This manuscript was prepared using the guidelines provided by Leisman et al. (31) for reporting of prediction models.

## Results

**Figure 2:**
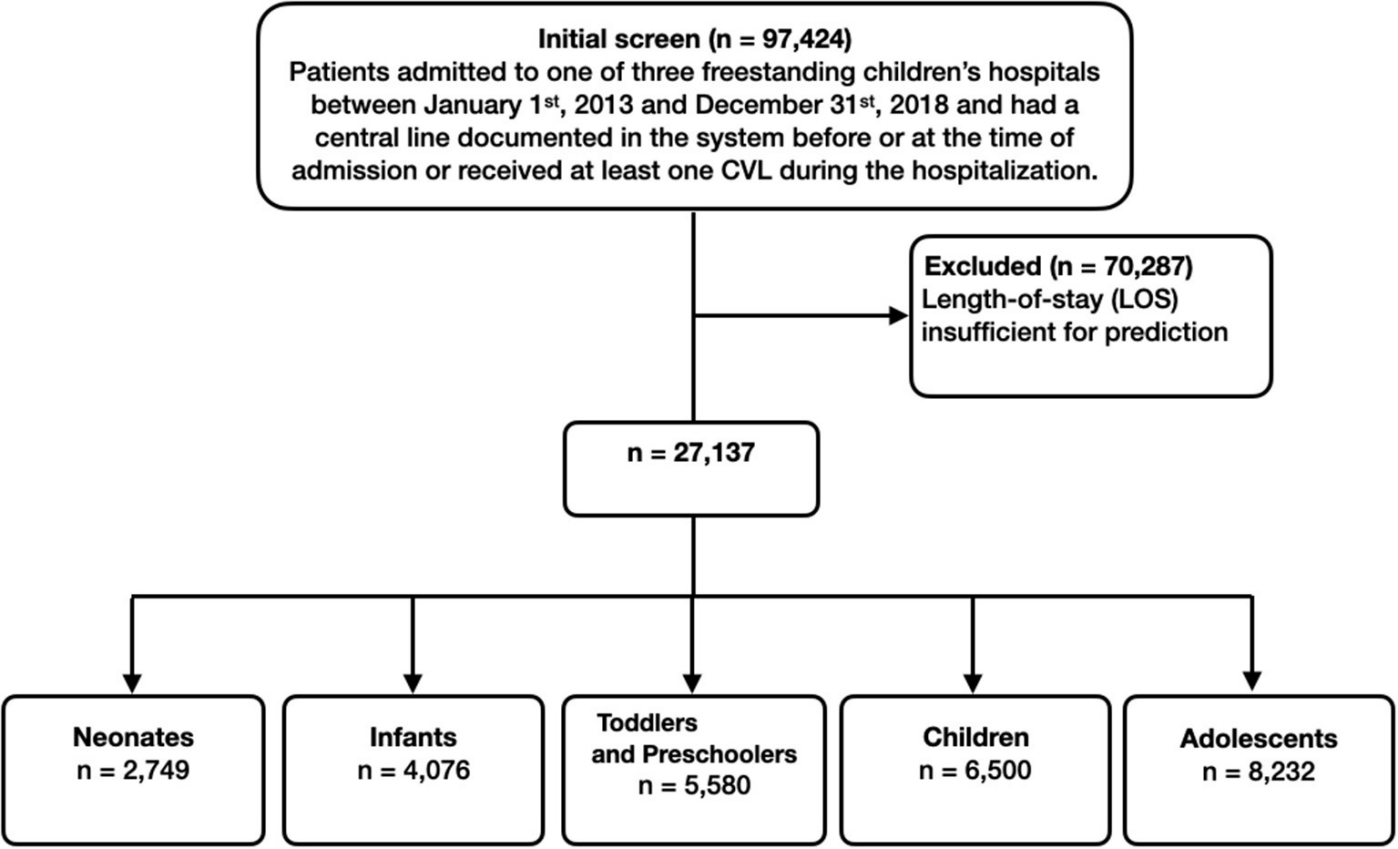
Inclusion flowchart. The final number of patient visits that we used in training and testing the machine learning models were 27,137.

In total, 97,424 patient encounters associated with 15,704 patients were extracted from the EHR. Of these, 70,287 encounters were excluded due to length-of-stay less than 24 h (most likely representing appointments for patients with existing CVLs). A total of 2,749 neonates (age less than 28 days), 4,076 infants (age between 28 days and one year), 5,580 toddlers and preschoolers (age between one and five years), 6,500 children (age between five and 12 years), and 8,232 adolescents (older than 12 years) met eligibility criteria. Figure 2 presents the associated CONSORT diagram.

Table 1 presents the cohort characteristics. There was a statistically significant difference between the median age, weight, and height with PSI^*^ patients younger and smaller. Length of stay was significantly longer in patients with PSI^*^African American race and Medicaid insurance were significantly more common in patients with PSI^*^. There was no statistically significant difference in gender between PSI^*^ and non- PSI^*^ groups. Moreover, statistical tests were performed to investigate if there were statistically significant differences between the components of PELOD-2 (Appendix C) and PRISM-III (Appendix D) between PSI^*^ and non-PSI^*^ groups across time windows.

**Table 1:** Cohort characteristics.

	**With PSI** ^*^	**Without PSI** ^*^	***p*-value**
Age (years) (Median [25th, 75th])	3.6^a^ [0.2, 12.6]	6.1 [1, 13.4]	<0.001
Weight (Kg) (Median [25th, 75th])	14.3^a^ [3.8, 40.9]	20 [8.5, 45.3]	<0.001
Height (cm) (Median [25th, 75th])	93^a^ [52, 149]	112 [69, 152]	<0.001
Length of Stay (LOS) in Days (Median [25th, 75th])	36^a^ [23, 69]	5 [3, 13]	<0.001
Gender			
Male (%)	44.8	45.5	0.737
Race Asian (%) Caucasian (%) African American (%) American Indian or Alaska Native (%) Native Hawaiian or Pacific Islander (%) Other (%)	4.1 46.7^a^ 43.8^a^ 0.4 0.2 4.7	3.9 54.3 35.9 0.2 0.2 5.5	0.796 <0.001 <0.001 0.292 0.992 0.465
Insurance status Commercial (%) Public - Medicaid (%) Public - non-medicaid (%) Self-pay (%)	34.5^a^ 62.1^a^ 2.9 0.39	39.4 56.6 3 0.9	0.025 0.013 0.933 0.224
ICU admission (%)	63.9^a^	44.7	<0.001
Placed on extracorporeal membrane oxygenation (%)	8.7^a^	2.1	<0.001
Mortality (%)	0.20	0.06	0.19

a Statistically significant difference between PSI^*^ and non-PSI^*^ groups.

**Figure 3:**
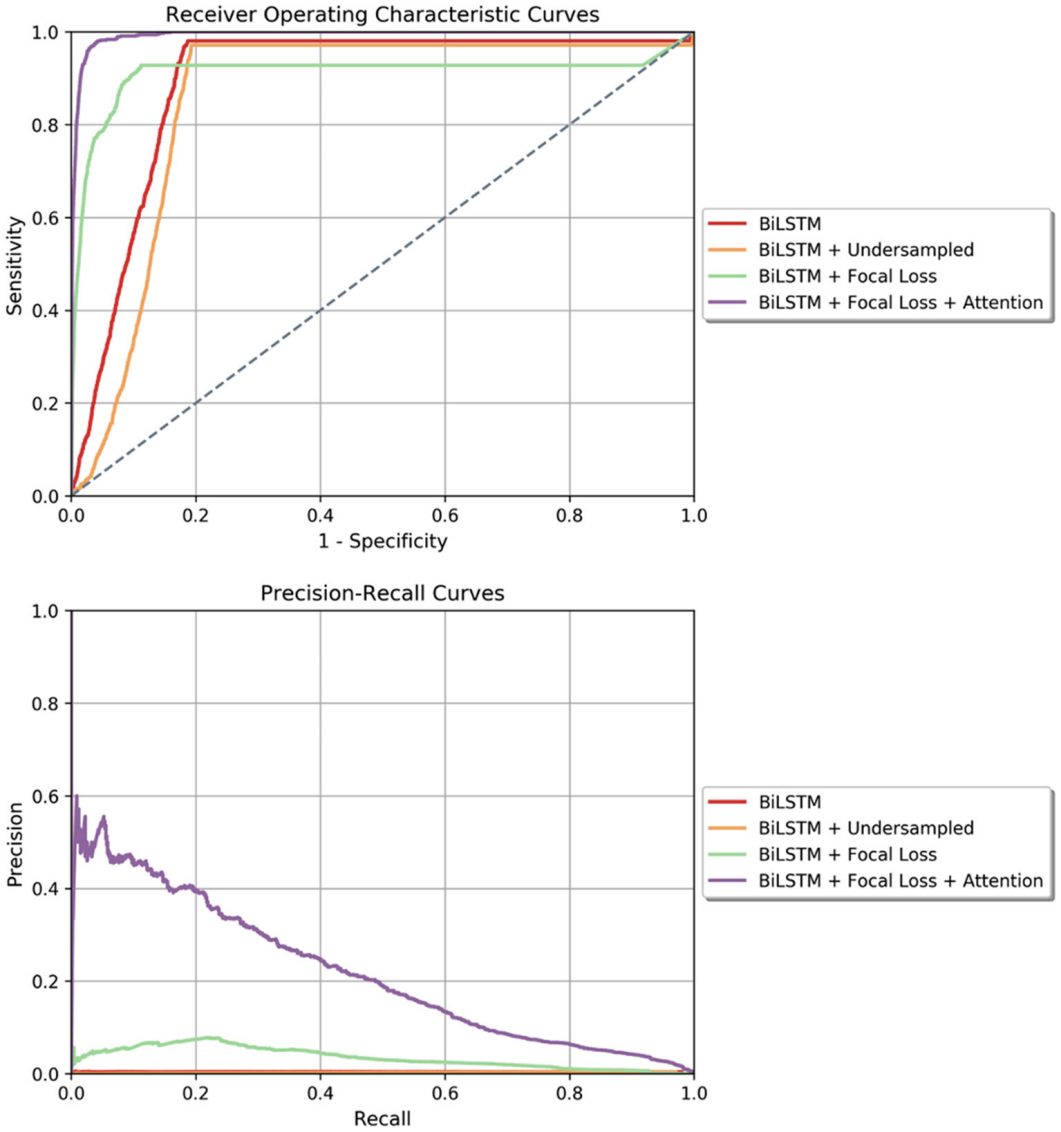
(Top) Receiver Operating Characteristics curves for all four models tested in the window-wise study. (Bottom) Precision-Recall curve for all the models tested in the window-wise study. In both plots, our proposed model which is the Bidirectional LSTM with Focal loss and attention mechanism achieved the highest area under curve.

The results of the four predictive models are presented in Table 2. Our proposed model, the Bidirectional LSTM with Focal loss and attention mechanism, outperformed the rest of the models with AUROC of 99.3% [99.0%, 99.6%] and AUPRC of 13.9% [10.6%, 18.0%]. The ROC and Precision-Recall curves of all trained models are presented in Figure 3. Fixing the sensitivity of all models to 85% to select a threshold, our proposed model's specificity was 99.4% [99.2%, 99.5%], F-1 was 9.9% [7.1%, 13.8%] and PPV was 7.7% [5.7%, 10.3%] which is 23 times the baseline PSI^*^ prevalence (0.34%). All performance metrics except for sensitivity and NPV were statistically different from the other models' metrics (*p* < 0.001). Moreover, the model generated 0.049 [0.044, 0.054] false alarms per patient per day. In other words, there should be 34 positive PSI^*^ per 10,000 48 h time windows (prevalence of 0.34%). The results of the proposed model indicated that per 10,000 predictions which lead to X number of positive predictions, 7.7% of X will be the number of PSI^*^ windows that were correctly predicted as positive. Besides, 99.9% of non-PSI^*^ windows were correctly predicted as negative ones. Moreover, 85% of true PSI^*^ were predicted correctly while 15% of the true PSI^*^ time windows were predicted as negative ones.

**Table 2:** Performance metrics of the deep learning models in predicting PSI^*^ in the next 48 h of hospitalization.

**Metrics**	**Bidirectional LSTM**		**Bidirectional LSTM** **+** **Under sampling majority class**		**Bidirectional LSTM** **+** **Focal loss**		**Bidirectional LSTM** **+** **Focal loss** **+** **Attention**	
	**Train**	**Test**	**Train**	**Test**	**Train**	**Test**	**Train**	**Test**
AUROC (%)	88.4 [86.7, 89.9]	89.3 [86.6, 91.5]	88.3 [86.8, 89.6]	85.7 [82.5, 88.2]	92.8 [90.8, 94.8]	91.1 [86.8, 94.9]	99.7 [99.6, 99.7]	99.3 [99.0, 99.6]
Sensitivity (%)	85.1 [85.0, 85.2]	85.3 [78.3, 91.6]	85.1 [85.0, 85.2]	81.1 [72.7, 88.4]	85.1 [85.0, 85.2]	83.3 [75.3, 90.2]	85.1 [85.0, 85.2]	72.9 [62.8, 82.1]
Specificity (%)	84.3 [83.6, 85.2]	84.2 [83.2, 85.2]	84.0 [83.3, 84.7]	83.2 [82.4, 83.9]	93.6 [92.5, 94.7]	93.2 [92.0, 94.3]	99.4 [99.2, 99.6]	99.4 [99.2, 99.5]
Positive Predictive Value (%)	0.4 [0.3, 0.4]	0.4 [0.3, 0.5]	0.4 [0.3, 0.4]	0.3 [0.3, 0.4]	1.0 [0.8, 1.2]	0.9 [0.7, 1.1]	9.4 [6.9, 12.5]	7.7 [5.7, 10.3]
Negative Predictive Value (%)	99.9 [99.9, 99.9]	99.9 [99.9, 99.9]	99.9 [99.9, 99.9]	99.9 [99.9, 99.9]	99.9 [99.9, 99.9]	99.9 [99.9, 99.9]	99.9 [99.9, 99.9]	99.9 [99.9, 99.9]
Accuracy (%)	84.3 [83.6, 85.2]	84.2 [83.2, 85.2]	84.0 [83.3, 84.7]	83.2 [82.4, 83.9]	93.5 [92.5, 94.7]	93.1 [92.0, 94.3]	99.4 [99.2, 99.6]	99.3 [99.2, 99.5]
F-1 Score (%)	0.8 [0.7, 0.9]	0.8 [0.6, 0.9]	0.8 [0.7, 0.9]	0.7 [0.6, 0.8]	3.3 [2.3, 4.2]	2.6 [1.4, 3.9]	58.0 [46.0, 70.8]	16.1 [10.4, 22.5]
AUPRC (%)	0.4 [0.3, 0.4]	0.4 [0.3, 0.5]	0.3 [0.3, 0.4]	0.3 [0.2, 0.3]	3.9 [3.1, 4.7]	3.2 [1.9, 5.3]	80.7 [76.3, 84.6]	41.2 [30.7, 50.2]

*The two numbers in the brackets present the estimated 95% confidence interval using bootstrap sampling. LSTM, long short-term memory; AUROC, area under receiver operating characteristic; AUPRC, area under precision-recall curve*.

### Explainability

**Figure 4:**
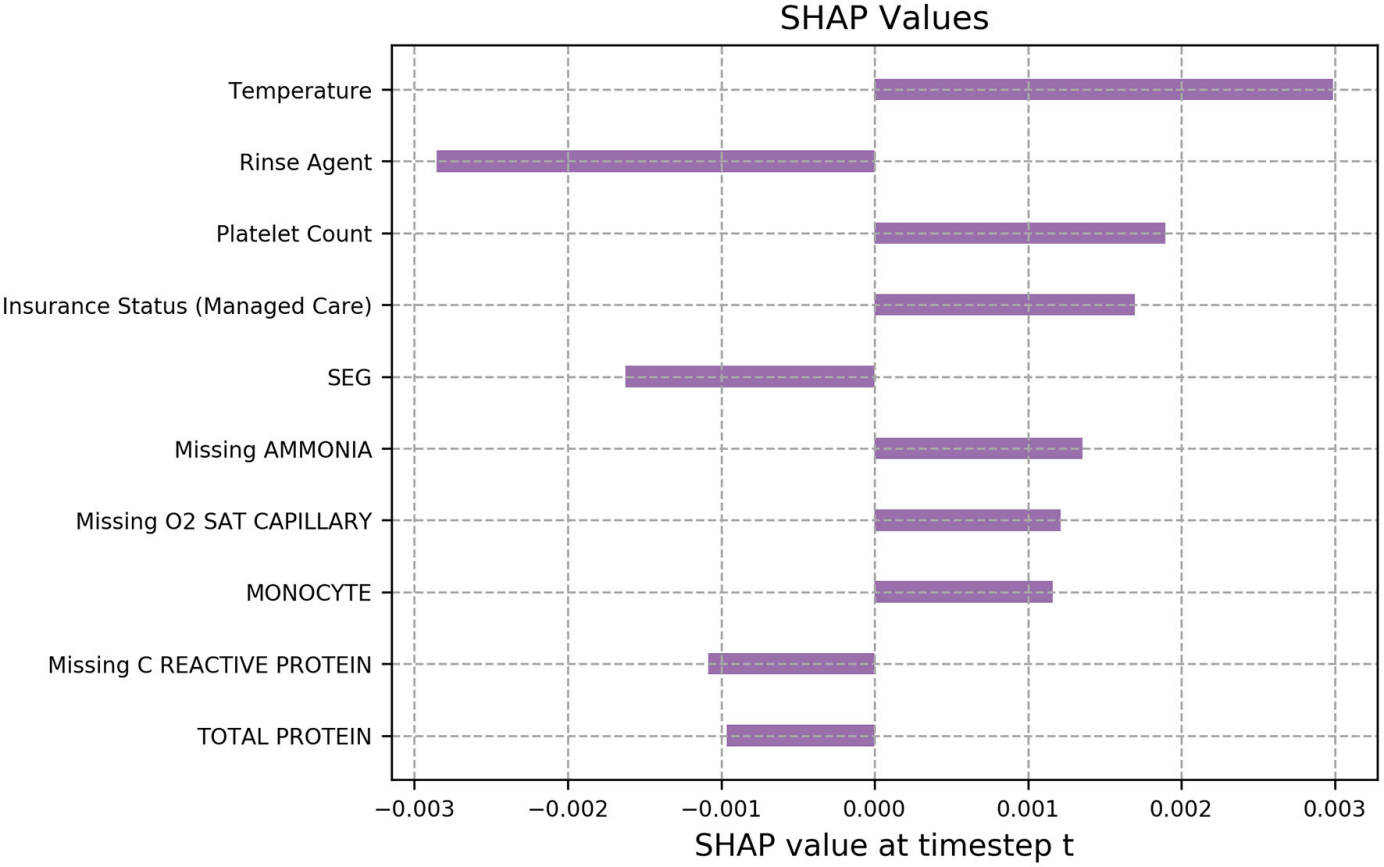
Feature importance plot based on SHAP values for an example prediction in which the model predicted the patient would develop a CLABSI within the next 48 h.

For the final model, we calculated SHAP values of each feature at every prediction point. Figure 4 presents the most important features for a specific timestamp in which the model predicted positive PSI^*^. For this patient, temperature had the highest effect size on the predicted outcome, followed by rinse agent, which was used to remove germs from the mouth, and platelet count.

### Comparison to PELOD-2

The performance of PELOD-2 in window-wise prediction of PSI^*^ is presented in Table 3. The cut-off points that yield higher performance metrics are listed. On the testing set, the best PPV was achieved at a cut-off point of PELOD-2 = 8 (1.5% [0.9%, 2.1%]) which was almost 5 times the baseline prevalence. At this cut-off value, the sensitivity was 3.2% [1.8%, 4.5%], specificity was 99.2% [99.2%, 99.3%], F-1 was 2% [1.2%, 2.9%]. Comparing to the proposed model, there were lower values achieved for PPV (6.2% drop), sensitivity (69.7% drop), F-1 (7.9% drop) but specificity of PELOD-2 model was almost similar to the proposed model.

**Table 3:** Performance of PELOD-2 score in predicting PSI^*^ in the next 48 h of hospitalization.

**Metrics**	**PELOD-2 Threshold** **=** **4**		**PELOD-2 Threshold** **=** **6**		**PELOD-2 Threshold** **=** **8**		**PELOD-2 Threshold** **=** **10**	
	**Train**	**Test**	**Train**	**Test**	**Train**	**Test**	**Train**	**Test**
Sensitivity (%)	46.0 [44.0, 47.8]	52.0 [48.4, 55.9]	6.6 [5.6, 7.6]	7.4 [5.5, 9.5]	2.0 [1.5, 2.6]	3.2 [1.8, 4.5]	0.7 [0.4, 1.0]	0.6 [0.0, 1.2]
Specificity (%)	83.2 [83.1, 83.2]	82.7 [82.5, 82.8]	97.2 [97.2, 97.3]	97.1 [97.0, 97.2]	99.2 [99.2, 99.2]	99.2 [99.2, 99.3]	99.8 [99.8, 99.8]	99.9 [99.8, 99.9]
Positive Predictive Value (%)	0.9 [0.9, 1.0]	1.1 [1.0, 1.2]	0.8 [0.7, 0.9]	0.9 [0.7, 1.2]	0.8 [0.6, 1.1]	1.5 [0.9, 2.1]	1.2 [0.6, 1.8]	1.5 [0.0, 3.1]
Negative Predictive Value (%)	99.8 [99.8, 99.8]	99.8 [99.8, 99.8]	99.7 [99.7, 99.7]	99.6 [99.6, 99.7]	99.7 [99.7, 99.7]	99.6 [99.6, 99.7]	99.7 [99.7, 99.7]	99.6 [99.6, 99.7]
Accuracy (%)	83.0 [82.9, 83.1]	82.5 [82.4, 82.7]	96.9 [96.9, 97.0]	96.8 [96.7, 96.9]	98.9 [98.9, 98.9]	98.9 [98.8, 98.9]	99.5 [99.5, 99.5]	99.5 [99.5, 99.5]
F-1 Score (%)	1.8 [1.7, 1.9]	2.1 [1.9, 2.4]	1.4 [1.2, 1.7]	1.7 [1.2, 2.1]	1.2 [0.9, 1.5]	2.0 [1.2, 2.9]	0.8 [0.5, 1.3]	0.9 [0.0, 1.8]

Different thresholds are selected for the PELOD-2 values. If a score exceeded the threshold, the predicted outcome for that patient would be developing PSI^*^ during the next 48 h of hospitalization. The two numbers in the brackets present the estimated 95% confidence interval using bootstrap sampling.

## Discussion

Many important clinical events where accurate predictions could improve outcomes such as sepsis, deterioration, or cardiac arrest are rare, especially in pediatrics (32–34). The prevalence of these conditions would be even lower if estimated over 48 h time intervals during hospitalization instead of only counting the final outcome over an entire hospital stay. The techniques described in this study would likely translate to prediction of other clinical events with extreme class imbalance.

We developed a novel algorithm to predict a presumed serious infection in a hospitalized pediatric patient within 2 hospital days. Besides having a decent predictive performance, our proposed model employed SHAP values which explained the effect of the salient features on the risk of a PSI^*^ event. Moreover, the SHAP values present the most influential features specific to a patient in a given time; therefore, these values can dynamically change through time as the condition of a patient changes. SHAP values give insight to the model's decision-making process by providing transparency and observability to the end-user of the features most important to model prediction. Insight into the model's focus for a specific prediction allows the end user to calibrate trust in the prediction.

Predictive models intended for use in clinical environments must recognize the complex adaptive systems in which they will be implemented (29, 35). The sensitivity and PPV of the model can inform the appropriate time in workflow where the model would be most useful. Our model demonstrates strong enrichment (i.e., the PPV is 23 times higher than the baseline prevalence of PSI^*^) while maintaining good sensitivity, but the PPV is nonetheless quite low – only 1 of every 13 predictions developed a PSI^*^ in the subsequent 48 h. This apparent low PPV is in large part due to the window-wise design which lowers the apparent prevalence of PSI^*^ relative to using an entire encounter as the unit of analysis. Thus, we anticipate this model would be more likely to be used as a non-interruptive monitoring system (e.g., displayed on patient lists) that can segregate out low-risk patients (NPV 0.999) while informing clinicians' estimate of the risk of PSI^*^ in order to make decisions about line maintenance and interventions. Similarly, the model could direct attention for teams reviewing vascular access across a unit or a hospital to improve the efficiency of PSI^*^ prevention efforts. We further investigated the performance of the model in predicting PSI which means relaxing the restriction on the laboratory result of the culture drawn. The results are presented in Appendix E.

In our study cohort, PSI^*^ was more common in African American patients and those with Medicaid insurance. While this analysis was not designed to describe disparities or their sources, this finding was nonetheless consistent with health disparities seen in adult sepsis patients (36). Model performance was not significantly different by patient race or insurance status (Appendix F). We also performed sensitivity analysis based on patient age and included the results in Appendix G.

Our study has important strengths and limitations. We also had several limitations. First, our data was associated with a single pediatric health system and may reflect the particular structure and patient mix of this setting. While we extracted EHR features expected to be available across systems, the external application of our model on other health systems may be biased. Nonetheless, limiting to structured EHR data likely reduces the technical barriers to implementation in a real-time system. Second, our model was developed and evaluated based on a retrospective cohort. While we attempted to simulate prospective implementation using a window-wise design, predictive performance may deteriorate when implemented in real time. Third, we have not evaluated how these predictions would supplement clinical decision-making when clinicians determine to remove a CVL or change their interventions. Thus, it is possible that implementation at this or even a higher level of predictive performance may not change outcomes. Fourth, we included patients with CVLs placed prior to admission. While inclusion of CVLs placed prior to admission may lower predictive performance since the model has fewer data available, we nonetheless felt it important to include as this reflects the decision-making clinicians must make in reality about all CVLs whether placed locally or not. Finally, we benchmarked our comparison vs. a standard of illness score. While not intended for the prediction of infections, PELOD, along with other scores such as the Pediatric Risk of Mortality (PRISM) and Pediatric Index of Mortality (PIM) scores are currently the only standard that exist to identify the risks of morbidity and mortality in hospitalized children. Thus, it would not be expected for these scores to have strong predictive performance for PSI^*^ associated with CVL. Nonetheless, we demonstrate our model's additional value when applied to this use case compared to existing severity scores.

We only included structured data in our analyses while unstructured data are known to have benefits when used in predictive models. Including text data or waveform data in subsequent iterations may improve our prediction outcomes.

## Conclusions

We developed a novel, explainable deep learning framework that can predict if PSI^*^ will occur for a patient with CVL during the next 48 h of hospitalization using routinely recorded features in EHR. This model provides insights to its decision making by providing the most influential features and their effect sizes on the predicted probability of PSI^*^ during the next 48 h of hospitalization. This framework is capable of being implemented in a real-time setting and serve as a clinical decision support system.

## Data Availability Statement

The data analyzed in this study is subject to the following licenses/restrictions: The dataset includes electronic health records for hospitalized patients at Children's Healthcare of Atlanta (CHOA) from 2013 to 2018 who had central lines. We cannot publicly share this data due to protected health information protocols. Requests to access these datasets should be directed to rkamaleswaran@emory.edu.

## Ethics Statement

This study was reviewed and approved by Emory University (protocol number 19-012).

## Author Contributions

AT performed experimental design, data processing, modeling, statistical analysis, article drafting, and article revision. EO and RK were involved with data collection, experimental design, and article revision. RB, SN, and GC were involved with article revision. All authors contributed to the article and approved the submitted version.

## Funding

This project was supported by an institutional grant provided by the Children's Healthcare of Atlanta, Atlanta, GA through The Pediatric Technology Center, in conjunction with the Health Analytics Council. AT was also funded by the Surgical Critical Care Initiative (SC2i), Department of Defense's Defense Health Program Joint Program Committee 6/Combat Casualty Care (USUHS HT9404-13-1-0032 and HU0001-15-2-0001). All views expressed in this article are the authors' own and do not necessarily reflect the views of the authors' employers and funding bodies.

## Conflict of Interest

In the last three years GC has received research funding from the NSF, NIH and LifeBell AI, and unrestricted donations from AliveCor, Amazon Research, the Center for Discovery, the Gordon and Betty Moore Foundation, MathWorks, Microsoft Research, the Gates Foundation, Google and the One Mind Foundation. GC has financial interest in AliveCor, and receives unrestricted funding from the company. GC also is the CTO of MindChild Medical and CSO of LifeBell AI and has ownership in both companies. These relationships are unconnected to the current work. The remaining authors declare that the research was conducted in the absence of any commercial or financial relationships that could be construed as a potential conflict of interest.

## Publisher's Note

All claims expressed in this article are solely those of the authors and do not necessarily represent those of their affiliated organizations, or those of the publisher, the editors and the reviewers. Any product that may be evaluated in this article, or claim that may be made by its manufacturer, is not guaranteed or endorsed by the publisher.
