# Supplementary material for: Deep Learning Model to Predict Serious Infection Among Children With Central Venous Lines
Source: Front Pediatr. 2021 Sep 15;9:726870. doi: 10.3389/fped.2021.726870 (PMC8480258; doi:10.3389/fped.2021.726870)
Supplement: Supplementary file 1 [file Data_Sheet_1.docx]

## **Supplement Material**

### Appendix A

##### A.1. Features

The complete list of the features extracted from Children’s Healthcare of Atlanta database is mentioned in the following. To preserve the models’ reproducibility, we selected the features that are most likely available across all healthcare systems; therefore, we incorporated patient and encounter level, central line property and microbiology information, current medications, antibiotic administration, laboratory results and measured vital signs.

Features extracted from CHOA clinical database:

- Patient-Level data: Name, DOB, MRN, Sex, Race, Ethnicity, Zip Code, Gestational Age, Birth Weight, Primary Language.
- Encounter-Level data: CSN, Admission Date/Time, Discharge Date/Time, Department(s), All diagnoses prior to admission date, All Diagnoses prior to discharge date, Admission Diagnoses, Hospital Diagnoses, Flags (Oncology Diagnoses, BMT Diagnoses, Transplant Diagnoses, Short Gut Syndrome, NEC, DiGeorge, SCID, Downs, Heterotaxy), Insurance status, Admission Weight, Admission Height, Caregiver cognitive factors including ability to read
- ICU Admission-Level data: Time of transfer into ICU, Time of transfer out of ICU, Flags (PICU, CICU, NICU, Technology-dependent ICU)
- Co-morbidities variable: Presence and number of pediatric complex care conditions (see R package or original citation)
- PRISM-3 at 1st PICU admission, PIM-2 at 1st PICU admission, SNAPPE-2 at NICU admission
- ECMO/Bypass variable: ECMO Start Time, ECMO End Time, Cardiac Bypass Start Time, Cardiac Bypass End Time
- Bone Marrow Transplant Level Data Variable: HCT Type, Transplant Date, Presence of Acute GVHD, Presence of Chronic GVHD
- Line Properties Variable: Line Insertion Date, Line Type, SITE Location, Gauge, Needle Length, Patient Prep, Insertion Bundle Complete?, Site Prep, Patient Tolerance, Inserted By, Discharged with Line/Drain/Tube, Removal Date/Time
- Line Timestamped Data Variable: Line/Site/Dressing WNL, Status, Line Exception, Line intervention, Needle Type, Needle -Manufacturer, Needle Gauge, Needle Length (decimal), Needle Length (fraction), Site exception, Site intervention, Dressing type, Dressing exception, Dressing intervention
- Endotracheal Tube Properties Variable: Placement Date, Placement Time, Airway Type, Size, Number of Attempts, Removal Date/Time
- Medication-Level Data Variable: Med name, Med dose, Med route, Med start date/time, Med stop date/time, Therapeutic Class, Pharmacy class, Pharmacy Subclass, Chemotherapy?, Antibiotic?, Fluid Bolus?, Dextrose Concentration if Fluid or TPN?, TPN?, Intralipid?, TPA for catheter clearance?, Blood Product? (PRBCs, Platelets, FFP, Cryo, Factor 8, G-CSF, IVIG), Sedation drip, Vasopressors/Inotropes, Systemic Steroid, Systemic Hydrocortisone, Systemic Immunosuppressant, Opioid pain medication, Paralytic, Diuretic, Insulin drip, Insulin intermittent
- Microbiology Data Variable: Culture Collection Date/Time, Culture Source (Specimen Description), Culture Source (Special Requests), Blood Culture Date/Time growth noted, Blood Culture Result – Gram Stain, Blood Culture Result – Species, Blood Culture Result – Susceptibilities, Respiratory Viral Panel, Respiratory Culture, Urine Culture, Stool PCR, Wound Culture, Eye culture
- Non-Micro Lab Data Variables: WBC, RBCS, HGB, HCT, MCV, MCH, MCHC, RDW, PLATELET COUNT, MEAN PLT VOLUME, AUTOMATED ABS NEUT, SEG, BAND, LYMPHOCYTE, MONOCYTE, METAMYELOCYTE, MYELOCYTE, Ammonia, Arterial pH, Arterial pO2, Arterial pCO2, Arterial O2 sat, Arterial Base Deficit, Venous pH, Venous pO2, Venous pCO2, Venous O2 sat, Venous Base Deficit, Capillary pH, Capillary pO2, Capillary pCO2, Capillary SaO2, Capillary Base Deficit, Lactate, Troponin, BNP, Cortisol, Na – lab, Na – gas, K – lab, K - gas, Cl – lab, HCO3 – lab, HCO3 – gas, BUN, Cr, BUN/Cr, Glucose - lab, Glucose - gas, Ca – lab, Ionized Ca – gas, Magnesium, Phosphorus, AST, ALT, Albumin, Total Protein, Alk Phos, GGT, Bilirubin, INR, PT, PTT, Anti-Xa, Fibrinogen, D-Dimer, AT3, Activated Clotting Time, CRP, ESR, CPK
- Presence and time of radiology studies: Chest X-ray, CT (any), Ultrasound (any), Abdominal X-Ray
- Immunization Data Variable: Immunizations received prior to hospital admission date, Immunizations received during hospital encounter
- Vital Signs Variable: Core Temperature, Temperature, Heart Rate, Respiratory rate, Arterial line BP, Non-invasive BP, SpO2, ET CO2, CVP, Capillary Refill
- Other Time-Stamped Data Variable: Tooth Brushing (Mouth Care), Rinse Agent, Bath (including CHG Bath), High touch surface clean protocol, Linen change, Patient Behaviors, Parent/Caregiver Behaviors, Parent/Caregiver Involvement, Braden Q score, CAPD scores, Current SBS (State Behavioral Score), Desired SBS, WAT score, NIRS Left, NIRS Right, Oxygen Mode, FiO2 (%), Oxygen Flow (lpm), Type of Mechanical Ventilation, CPAP Pressure, Bilevel Pressures (IPAP/EPAP), Oxygenation Index, PaO2/FiO2, Type of Mechanical Ventilation, Ventilator Mode, Ventilator Rate, Set PIP, Measured PIP, PEEP, Tidal Volume Set, Tidal Volume Exhaled, Mean Airway pressure, Minute Ventilation, Spontaneous Rate, Inspiratory Time, Rise Time (slope), Pressure Support, Sensitivity, APRV Pressure High, APRV Pressure Low, APRV Time High, APRV Time Low, APRV Ventilator Rate, APRV Mean Airway Pressure, APRV Dump Volume, APRV Minute Volume, HFOV Hertz, HFOV DeltaP/Amplitude, HFOV Mean Airway Pressure, HFOV Inspiratory Time (%), Nitric Oxide Start/Stop, Inhaled Nitric Oxide, Urine Output (mL), Urinary Frequency, Bladder Scan, Emesis, Emesis (Frequency), Stool (measured), Stool frequency, Bladder Pressure, Output CVVH Ultrafiltrate, HD Positive Ultrafiltrate, HD Negative Ultrafiltrate, PD Positive Ultrafiltrate, PD Negative Ultrafiltrate, Time on, Time off, Time of order placement, Procedure name, Procedure start time, Procedure end time, Mucus fistula placement date, Fluoroscopy of mucus fistula.

##### A.2. Data Preprocessing

At the time of the study, the data from 2013 to 2018 was provided by the institution, so we could not acquire 2019 and 2020 data.

After gathering all the windows from the patients’ hospitalization, the data was preprocessed and ready for model training. Initially, there were 252 features in the data. No information from discharge was passed to the input of the predictive model. The following preprocessing steps were done:

- Data capping: to reduce the effect of the outliers, 0.5 and 99.5 percentiles of each feature were calculated and set as the upper and lower bounds for the values. Any value above the upper bound was replace with the upper bound and any value below the lower bound was replaced with the lower bound.
- Transformation: the numerical variables were log or square root transformed to push their distribution more towards the normal distribution.
- Imputation: the missing values were imputed with the median value of the corresponding feature.
- Standardization: each feature was scaled by subtracting the corresponding feature’s mean value and dividing by the associated standard deviation.

We applied one-hot-encoding to the binary and categorical variables, to measure distinct aspects of these variables and to ensure that these categorical variables are appropriately distinguished from continuous measures. Missing laboratory tests could not be at random, so they may convey information (1). To capture that information, we added a binary flag for each laboratory value. The flag is one when the value is missing and zero otherwise.

To remove collinearity, we set a threshold of 0.8 for pair-wise correlation among features. If the pairwise correlation between two features exceeded the threshold, we removed one of them and kept the other one in the input. After removing collinearity with the threshold of 0.8, the feature space reduced to 135 dimensions. The selected features are listed in the following.

Laboratory Results

O2 Saturation Capillary, O2 Saturation Venous, Albumin, Alkaline Phosphatase, ALT SGPT, Ammonia, Antithrombin Assay, Arterial Base Excess, Arterial POC PCO2, Arterial POC PH, Arterial POC PO2, AST SGOT, Atypical Reactive Lymphocyte, Automated Absolute Neutrophil, BAND, Bilirubin Total, BNP, Blood Urea Nitrogen, C-Reactive Protein, Calcium, CAP Base Deficit, CAP Base Excess, Capillary POC PCO2, Capillary POC PH, Capillary POC PO2, Chloride, Cortisol, Creatine Phosphokinase, Creatinine, D-dimer Units, Eosinophils, Erythrocyte Sedimentation Rate, Fibrinogen, Gamma GGT, Glucose, HCO3, Hemoglobin, International Normalized Ratio, Magnesium, MCH, MCHC, MCV, Mean Platelet Volume, Metamyelocyte, Monocyte, Myelocyte, Phosphorus, Platelet Count, POC Calcium Ionized, POC Glucose, POC Lactic Acid, POC Potassium, POC Sodium, Potassium, PTT, Red Cell Distribution Width, SEG, Sodium, Total Protein, Troponin, Venous POC PCO2, Venous POC PH, White Blood Cells, Missing Saturation Capillary, Missing O2 Saturation Venous, Missing Albumin, Missing Ammonia, Missing Arterial Base Excess, Missing Atypical Reactive Lymphocyte, Missing Automated Absolute Neutrophil, Missing BAND, Missing BNP, Missing C-Reactive Protein, Missing D-dimer Units, Missing Eosinophils, Missing Fibrinogen, Missing HCO3, Missing Magnesium, Missing Metamyelocyte, Missing Phosphorus, Missing POC Lactic Acid

Vital Signs

Core Temperature, Temperature, Heart Rate, Respiratory rate, Arterial line BP, SpO2, ET CO2, CVP, Systolic BP, Diastolic BP, Capillary Refill, GCS, PaO2/FiO2

Mechanical Ventilation

Was the patient on mechanical ventilation?

Demographics

Age < 28 days, 29 days < Age < 1 year, 1 year < Age < 4 year, 5 year < Age < 11 year, Age > 12 years, Gestational Age, Birth Weight, Gender, Race (Asian), Race (White), Race (Black or African American), Race (American Indian or Alaska Native), Race (Native Hawaiian or Other Pacific Islander), Ethnicity (Hispanic or Latino), Ethnicity (Non-Hispanic or Latino), Admission Weight, Insurance Status (CMO Medicaid), Insurance Status (Commercial), Insurance Status (Managed Care), Insurance Status (Medicaid), Insurance Status (Medicare), Insurance Status (Out of State Medicaid), Insurance Status (Self-pay), Insurance Status (Shared Service), Insurance Status (Tricare), Caregiver Cognitive Factors Flag

Medications

Chemotherapy, Fluid Bolus, TPN, Intralipid, Sedation Drip Grouper, Sedation Drip Bolus, Vasopressors Inotropes Grouper, Systemic Steroid Grouper, Opioid pain medication, Diuretic, Rinse Agent, Antimicrobial Bath

Line Properties and Line Insertion Information

Gauge, Line Type (Apheresis Port Dual)

### Appendix B

All the models and analysis were performed in Python 3.6.

#### Training/Validation/Testing Data Splits

To avoid data leakage, we split the data to train and test sets based on patient encounters; therefore, if a patient encounter was selected to be in the training set cohort, there were no information leakage to the testing set.

The PSI* prevalence across all 48-hour time windows was 0.34% which implied an extreme class-imbalanced classification problem. To preserve the same prevalence in training and testing sets, we used stratified sampling method to split data to training set (80%) and testing set (20%). There were multiple hyperparameters in our model which required to be optimized. So, we split the training patient encounters to 90% and 10% subsets using stratified sampling and incorporated the smaller set as the validation cohort in the hyperparameter optimization process.

#### The Input Structure

We employed a bidirectional Long Short-term Memory (LSTM) model to predict if there would be a PSI* event during the next 48 hours of hospitalization. We aimed to use the model every 8 hours to reflect the shift change; therefore, we need the inputs to be sequences of feature values which was gathered every 8 hours during a patient’s hospitalization time.

The Bidirectional LSTM model takes a 3-dimensional input in this format:

(number of patient encounters, number of timesteps, number of features)

Each patient had a specific number of windows for prediction as this number increased with higher length-of-stay. By default, the number of features is fixed but the number of timesteps can vary. But the model needs a fixed value for the second dimension. To set the number of timesteps, we considered the maximum number of timesteps that a patient had in our training cohort which was 168. To have the same sequence length, we zero padded the information of the patients who had less that 168 timesteps in their hospital stay. LSTM-based models are designed in a way that they can skip these padded timesteps so that it will not hurt the model’s outcome.

#### Model Specifications

The following figure presents the proposed model structure. We trained a bidirectional LSTM model with Focal loss and attention mechanism which used a batch size of 128. The hyperparameters of the model were optimized by employing Bayesian optimization method with 100 epochs and an early stopping criterion if there was no improve in the model’s performance after 5 iterations. A list of the hyperparameters along with their optimized value are listed in the following. We did not change the default parameters of Focal loss (alpha=0.25, gamma=2).

- Adam optimizer with learning rate = 0.1
- Dropout regularization = 0.5
- Number of hidden units in the bidirectional LSTM model = 512
- Number of hidden units in the unidirectional LSTM model = 8
- Hidden units of the dense layer prior to the classification layer = 8

A sigmoid layer was added for the final classification task.


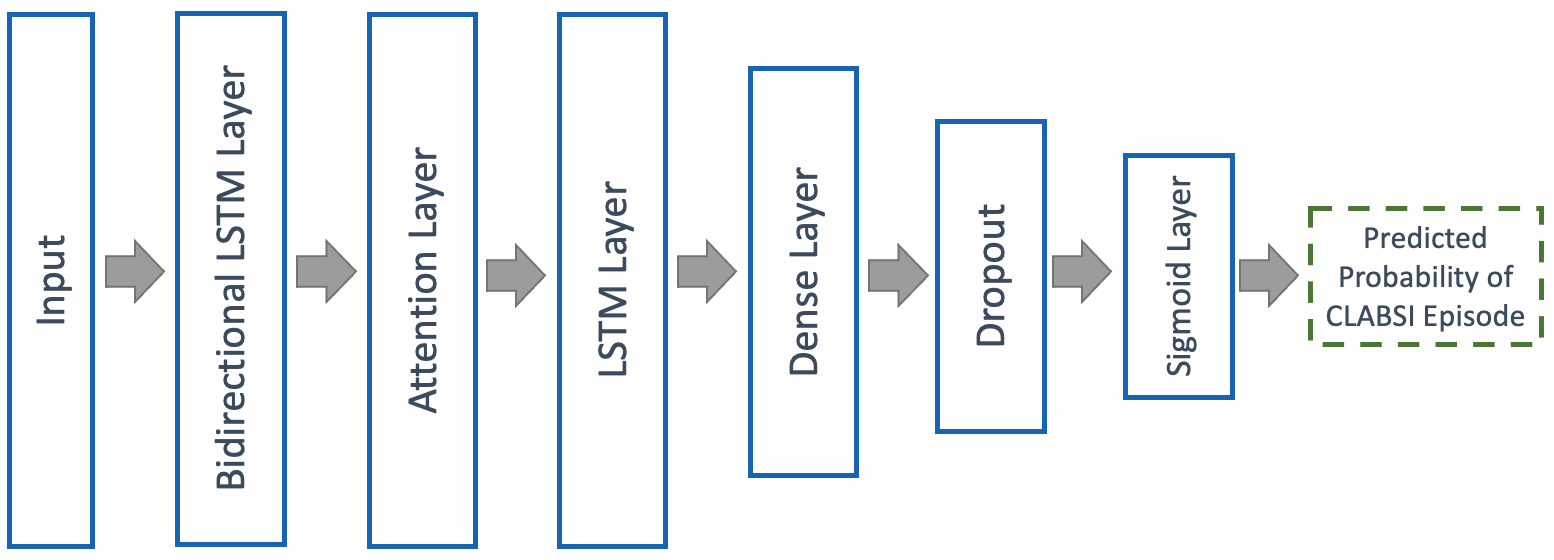


Figure B-1. The proposed model structure.

#### Confidence Interval of Performance Metrics

Bootstrap method with 1000 repetition was used to calculate an estimation of the 95% confidence interval for the performance metrics.

### Appendix C

PELOD-II score components

|  | **Time windows with PSI*** | **Time windows without PSI*** | **p-value** |
| --- | --- | --- | --- |
| **Glasgow coma score (Mean [SD])** | 11.9 [3.7] | 12.3 [3.6] | < 0.001 |
| **Lactatemia (mmol/L) (Mean [SD])** | 4.1 [7.1] | 2.2 [4.4] | < 0.001 |
| **Mean arterial pressure (mmHg)**  **Age in month <1 (Mean [SD])**  **1 < Age in month < 11 (Mean [SD])**  **12 < Age in month < 23 (Mean [SD])**  **24 < Age in month < 59 (Mean [SD])**  **60 < Age in month < 143 (Mean [SD])**  **Age in month >= 144 (Mean [SD])** | 71.9 [11.7]  75 [19.4]  74.7 [30.7]  72.2 [26.9]  85.7 [18.2]  111.1 [23.3] | 68.9 [15]  68 [18]  66.4 [20.8]  74.6 [20.5]  86.9 [19.7]  107.7 [23.7] | 0.026  < 0.001  0.026  0.311  0.559  0.008 |
| **Creatinine (μmol/L)**  **Age in month <1 (Mean [SD])**  **1 < Age in month < 11 (Mean [SD])**  **12 < Age in month < 23 (Mean [SD])**  **24 < Age in month < 59 (Mean [SD])**  **60 < Age in month < 143 (Mean [SD])**  **Age in month >= 144 (Mean [SD])** | 0.34 [0.23]  0.49 [0.41]  0.32 [0.14]  0.39 [0.47]  0.24 [0.09]  0.58 [1.02] | 0.38 [0.36]  0.49 [0.66]  0.37 [0.31]  0.31 [0.26]  0.27 [0.14]  0.58 [0.97] | 0.108  0.912  0.259  < 0.001  0.040  0.796 |
| **PaO2/FiO2 (mmHg) (Mean [SD])** | 25.6 [61.9] | 34 [103.7] | 0.002 |
| **PaCO2 (mmHg) (Mean [SD])** | 47.7 [10.7] | 45 [9.6] | < 0.001 |
| **Invasive ventilation (% Yes)** | 38.1 | 34.1 | 0.002 |
| **WBC (10^9^/L) (Mean [SD])** | 8.2 [9.2] | 10.4 [8.2] | < 0.001 |
| **Platelet (10^9^/L) (Mean [SD])** | 166.8 [154.7] | 295.3 [182.9] | < 0.001 |

Table C-1. This table presents the mean and standard deviation of PELOD-2 score components. The comparisons were done across the time windows. T-test and chi-square test were applied to test if there was a statistically significant difference between the feature values in windows with PSI* and windows without PSI* in patients’ hospitalization time.

### Appendix D

PRISM-III score’s components

|  | **With PSI*** | **Without PSI*** | **p-value** |
| --- | --- | --- | --- |
| **Systolic Blood Pressure (mm Hg)**  **Infants (Mean [SD])**  **Children (Mean [SD])** | 92.8 [16.9]  104.7 [13.4] | 93.1 [15.8]  105.8 [13.8] | 0.633  0.013 |
| **Diastolic Blood Pressure (mm Hg)  (Mean [SD])** | 57.2 [14.6] | 57.6 [15] | 0.128 |
| **Heart Rate (beats per minute)**  **Infants (Mean [SD])**  **Children (Mean [SD])** | 143 [24]  115 [23] | 135 [22]  106 [22] | < 0.001  < 0.001 |
| **Respiratory Rate (breaths per minute)**  **Infants (Mean [SD])**  **Children (Mean [SD])** | 39.8 [14.8]  24.1 [7.6] | 37.3 [13.7]  22.8 [6.3] | < 0.001  < 0.001 |
| **PaO2/FiO2 (Mean [SD])** | 25.6 [61.9] | 34 [103.7] | 0.002 |
| **PaCO2 in torr (mm Hg) (Mean [SD])** | 47.7 [10.7] | 45 [9.6] | < 0.001 |
| **Glasgow Coma Score (Mean [SD])** | 11.9 [3.7] | 12.3 [3.6] | < 0.001 |
| **PT/PTT (Mean [SD])** | 0.43 [0.12] | 0.44 [0.11] | 0.553 |
| **Total bilirubin (mg/dL) (Mean [SD])** | 2 [3.9] | 1.6 [2.9] | < 0.001 |
| **Potassium (mEq/L) (Mean [SD])** | 4 [0.69] | 4.1 [0.72] | < 0.001 |
| **Calcium (mg/dL) (Mean [SD])** | 8.7 [0.8] | 8.9 [0.8] | < 0.001 |
| **Glucose (mg/dL) (Mean [SD])** | 104.8 [37.4] | 103.1 [37.9] | 0.021 |
| **Bicarbonate in (mEq/L) (Mean [SD])** | 27.9 [5.9] | 27.1 [5.4] | < 0.001 |

Table D-1. This table presents the mean and standard deviation of PRISM-lll score components. The comparisons were done across the time windows. T-test was applied to test if there is a statistically significant difference between the mean of these features in windows with PSI* and windows without PSI* in patients’ hospitalization time.

### Appendix E

We incorporated PSI*, defined as a positive culture followed by at least four days of new antimicrobial agent administration, as the outcome because we were looking for a surrogate for CLABSI which does not need extensive chart reviews and can be extracted from EHRs.

Initially, we selected PSI as the outcome, which is a blood culture, regardless of the result, followed by at least four days of new antibiotic administration. After that, during the chart reviews to confirm the onset of the infection, we identified many false positives that were reduced by only considering the cultures associated with a positive result. Table E-1 in the following presents the performance of the deep learning model on the study cohort. The reason for achieving better performance metric values as higher prevalence of PSI (3.2%) comparing to PSI* (0.34%). It worth mentioning again that selecting PSI as the outcome generated many false positive alarms in predicting the infection; therefore, while the results are improved, using PSI* is a more reliable approach to predict the infection.

|  | Bidirectional LSTM +  Focal Loss + Attention | |
| --- | --- | --- |
|  | Train | Test |
| AUROC^a^ (%) | 97.7  [97.5, 97.9] | 97.0  [96.6, 97.4] |
| Sensitivity (%) | 85.0  [83.8, 86.2] | 80.3  [77.6, 82.9] |
| Specificity (%) | 95.6  [95.5, 95.8] | 95.5  [95.3, 95.7] |
| Positive Predictive Value (%) | 38.2  [36.9, 39.3] | 36.0  [33.8, 38.2] |
| Negative Predictive Value (%) | 99.5  [99.5, 99.6] | 99.4  [99.3, 99.5] |
| Accuracy (%) | 95.3  [95.2, 95.4] | 95.1  [94.8, 95.3] |
| F-1 Score (%) | 52.7  [51.4, 53.8] | 49.7  [47.4, 52.0] |
| AUPRC^b^ (%) | 71.7  [70.2, 73.3] | 63.5  [60.1, 66.6] |

^a^AUROC = area under receiver operating characteristic curve, ^b^AUPRC = area under precision-recall curve

Table E-1. This table presents the performance of the deep learning model in predicting PSI defined as blood culture regardless of the result followed by at least four days of new antibiotics administration. The numbers in the brackets are the estimated 95% confidence interval calculated by bootstrapping method.

### Appendix F

##### F.1. Model performance on different patient race categories

Patients with race category of white

- Training set: PSI* prevalence = 0.31%, number of encounters = 11894, number of 48-hour windows = 292465
- Testing set: PSI* prevalence = 0.35%, number of encounters = 2917, number of 48-hour windows = 91717

Patients with race category of black

- Training set: PSI* prevalence = 0.4%, number of encounters = 7707, number of 48-hour windows = 223528
- Testing set: PSI* prevalence = 0.36%, number of encounters =1951, number of 48-hour windows = 71937

Patients with race category of other

- Training set: PSI* prevalence = 0.3%, number of encounters = 2108, number of 48-hour windows = 51565
- Testing set: PSI* prevalence = 0.41%, number of encounters = 560, number of 48-hour windows = 17168

|  | Race (White) | | Race (Black) | | Race (Other) | |
| --- | --- | --- | --- | --- | --- | --- |
|  | Train | Test | Train | Test | Train | Test |
| AUROC^a^  (%) | 99.6  [99.5, 99.7] | 99.5  [99.3,  99.6] | 99.5  [99.4,  99.7] | 99.4  [99.1,  99.6] | 99.7  [99.4,  99.9] | 98.7  [96.4,  99.9] |
| Sensitivity  (%) | 84.3  [79.5, 88.5] | 76.1  [66.2,  85.5] | 85.4  [80.2,  90.2] | 77.9  [66.4,  87.8] | 85.8  [73.9,  96.1] | 75.6  [51.3,  97.1] |
| Specificity  (%) | 99.3  [99.3, 99.4] | 99.3  [99.3,  99.4] | 99.1  [99.1,  99.2] | 99.1  [99.0,  99.1] | 99.4  [99.3,  99.4] | 99.4  [99.3,  99.5] |
| Positive Predictive Value  (%) | 7.1  [6.6,  7.6] | 7.0  [6.0,  8.1] | 8.1  [7.6,  8.7] | 6.3  [5.2,  7.4] | 6.9  [5.6,  8.2] | 8.9  [5.8,  11.9] |
| Negative Predictive Value  (%) | 99.9  [99.9, 99.9] | 99.9  [99.9,  99.9] | 99.9  [99.9,  99.9] | 99.9  [99.9,  99.9] | 99.9  [99.9,  99.9] | 99.9  [99.9,  99.9] |
| Accuracy  (%) | 99.3  [99.3, 99.4] | 99.3  [99.3,  99.3] | 99.1  [99.1,  99.2] | 99.1  [99.0,  99.1] | 99.3  [99.3,  99.4] | 99.4  [99.3,  99.5] |
| F-1 Score  (%) | 13.0 [12.2, 13.9] | 12.9  [11.0,  14.7] | 14.8  [13.8,  15.8] | 11.7  [9.7,  13.6] | 12.7  [10.5,  15.0] | 15.9  [10.7,  21.1] |
| AUPRC^b^  (%) | 34.5  [27.4, 42.2] | 20.2  [12.4,  29.6] | 36.4  [29.4,  43.3] | 21.2  [12.2,  32.3] | 46.8  [30.8,  63.5] | 43.4  [18.1,  67.7] |

^a^AUROC = area under receiver operating characteristic curve, ^b^AUPRC = area under precision-recall curve

Table F-1. This table presents the performance of the proposed model on patients with different race categories in training and testing subsets of the data.

A one-way ANOVA test was performed and achieved a p-value of 0.29 for training and 0.86 for testing datasets. The results indicated no statistically significant difference between the mean of the model’s predicted probabilities for each race category.

##### F.2. Model performance on different patient insurance categories

Patients with Commercial insurance

- Training set: PSI* prevalence = 0.34%, number of encounters = 8772, number of 48-hour windows = 200683
- Testing set: PSI* prevalence = 0.37%, number of encounters = 2105, number of 48-hour windows = 60620

Patients with Public-Medicaid insurance

- Training set: PSI* prevalence = 0.35%, number of encounters = 12078, number of 48-hour windows = 347905
- Testing set: PSI* prevalence = 0.35%, number of encounters = 3124, number of 48-hour windows = 114446

Patients with Public-Medicare insurance

- Training set: PSI* prevalence = 0.41%, number of encounters = 665, number of 48-hour windows = 15407
- Testing set: PSI* prevalence = 0.27%, number of encounters = 156, number of 48-hour windows = 4707

Patients with Self-pay insurance

- Training set: PSI* prevalence = 0.19%, number of encounters = 194, number of 48-hour windows = 3563
- Testing set: PSI* prevalence = 0.64%, number of encounters = 43, number of 48-hour windows = 1049

|  | Insurance Status:  Commercial | | Insurance Status:  Public-Medicaid | | Insurance Status:  Public-Medicare | | Insurance Status:  Self-pay | |
| --- | --- | --- | --- | --- | --- | --- | --- | --- |
|  | Train | Test | Train | Test | Train | Test | Train | Test |
| AUROC^a^  (%) | 99.6 [99.4, 99.7] | 99.4  [98.7, 99.8] | 99.6 [99.5, 99.7] | 99.3 [99.1, 99.5] | 99.8 [99.6, 99.9] | 98.9 [97.8, 99.9] | 99.9  [99.9, 99.9] | 99.9  [99.8, 99.9] |
| Sensitivity  (%) | 80.1 [73.8, 85.7] | 79.1  [69.1, 88.8] | 87.4 [83.0, 91.6] | 75.6 [65.9, 84.2] | 89.8 [77.0, 99.9] | 55.3 [14.3, 99.9] | 99.9  [99.9, 99.9] | 99.9  [99.9, 99.9] |
| Specificity  (%) | 99.4 [99.4, 99.5] | 99.4  [99.4,  99.5] | 99.1 [99.1, 99.2] | 99.1 [99.0, 99.1] | 99.4 [99.3, 99.5] | 99.4 [99.2, 99.6] | 99.5 [99.4, 99.7] | 99.7 [99.4, 99.9] |
| Positive Predictive Value  (%) | 8.1  [7.3,  8.9] | 8.6  [7.3,  9.9] | 7.2  [6.7, 7.6] | 6.1  [5.2,  6.9] | 9.7  [7.6, 11.8] | 4.4  [1.1,  8.9] | 5.5  [4.0,  7.6] | 25.0 [14.3, 46.7] |
| Negative Predictive Value  (%) | 99.9  [99.9, 99.9] | 99.9  [99.9, 99.9] | 99.9  [99.9, 99.9] | 99.9  [99.9, 99.9] | 99.9 [99.9, 99.9] | 99.9  [99.9, 99.9] | 99.9  [99.9, 99.9] | 99.9  [99.9, 99.9] |
| Accuracy  (%) | 99.4 [99.4, 99.5] | 99.4  [99.4, 99.5] | 99.1 [99.1, 99.2] | 99.1 [99.0, 99.1] | 99.4 [99.3, 99.5] | 99.4 [99.2, 99.6] | 99.5 [99.4, 99.7] | 99.7 [99.4, 99.9] |
| F-1 Score  (%) | 14.7 13.3, 16.1] | 15.5  [13.3, 17.8] | 13.2 [12.5, 14.0] | 11.3  [9.6, 12.8] | 17.5 [14.0, 21.1] | 8.2  [2.0, 16.3] | 10.5  [7.7, 14.1] | 39.4 [25.0, 63.6] |
| AUPRC^b^  (%) | 36.0 [27.4, 44.6] | 21.6  [13.4, 32.0] | 37.2 [30.6, 43.5] | 24.5 [16.1, 34.4] | 31.7 [15.6, 53.4] | 7.8  [1.6, 25.4] | 73.6 [62.2, 90.7] | 49.7 [30.2, 90.2] |

^a^AUROC = area under receiver operating characteristic curve, ^b^AUPRC = area under precision-recall curve

Table F-2. This table presents the performance of the proposed model on patients with different insurance status in training and testing subsets of the data.

A one-way ANOVA test was performed and achieved a p-value of 0.09 for training and 0.13 for testing datasets. The results indicated no statistically significant difference between the mean of the model’s predicted probabilities for each insurance status category.

### Appendix G

Table G-1 in the following presents the performance of the proposed model on different patients’ age categories. These categories were defined as:

- Neonates: age < 28 days
- Infants: 29 days < age < 1 year
- Toddlers and Preschoolers: 1 year < age < 4 years
- Children: 5 years < age < 11 years
- Adolescents: 12 years < age

In deep learning models, as the positive cases increase, we can expect higher PPV value which is the same story in our analysis. In this study cohort, the prevalence of PSI* was slightly different across the five aforementioned age categories which influenced some of the performance metrics. Specifically, PPV and F-1 score increased as the PSI* prevalence increased. On the other hand, NPV, which is the power of the model to rule out the negative cases, was not affected by the changes in the prevalence as there were sufficient NP cases for the model to learn.

|  | Neonates | | Infants | | Toddlers and Preschoolers | | Children | | Adolescents | |
| --- | --- | --- | --- | --- | --- | --- | --- | --- | --- | --- |
|  | Train | Test | Train | Test | Train | Test | Train | Test | Train | Test |
| PSI* (%) | 0.32 | 0.27 | 0.39 | 0.27 | 0.42 | 0.44 | 0.32 | 0.38 | 0.30 | 0.45 |
| AUROC^a^  (%) | 99.4  [99.2, 99.6] | 99.4  [99.1, 99.6] | 99.5  [99.3, 99.6] | 99.3  [98.8, 99.7] | 99.7  [99.5, 99.8] | 99.5  [99.2, 99.8] | 99.7  [99.6, 99.9] | 99.5  [99.2, 99.8] | 99.6  [99.4, 99.7] | 99.2  [98.5, 99.7] |
| Sensitivity  (%) | 94.9  [91.3, 97.7] | 92.9  [82.3, 99.9] | 87.1  [80.0, 93.1] | 84.1  [67.0, 96.8] | 79.1  [70.4, 87.1] | 75.2  [59.7, 89.7] | 86.0  [77.3, 93.7] | 68.8  [48.3, 86.3] | 77.9  [69.3, 85.4] | 69.6  [56.3, 83.0] |
| Specificity  (%) | 98.1  [97.9, 98.2] | 98.0  [97.9, 98.2] | 98.8  [98.7, 98.8] | 98.7  [98.6, 98.8] | 99.5  [99.5, 99.5] | 99.5  [99.5, 99.6] | 99.5  [99.5, 99.5] | 99.4  [99.4, 99.5] | 99.6  [99.6, 99.6] | 99.5  [99.5, 99.6] |
| Positive Predictive Value  (%) | 6.5  [6.1, 6.9] | 5.4  [4.6, 6.1] | 7.1  [6.4, 7.7] | 4.6  [3.7, 5.4] | 9.3  [8.2, 10.4] | 10.1  [7.8, 12.5] | 7.4  [6.5, 8.2] | 6.5  [4.5, 8.4] | 8.3  [7.1, 9.4] | 9.7  [7.6, 11.7] |
| Negative Predictive Value  (%) | 99.9  [99.9, 99.9] | 99.9  [99.9, 99.9] | 99.9  [99.9, 99.9] | 99.9  [99.9, 99.9] | 99.9  [99.9, 99.9] | 99.9  [99.9, 99.9] | 99.9  [99.9, 99.9] | 99.9  [99.9, 99.9] | 99.9  [99.9, 99.9] | 99.9  [99.9, 99.9] |
| Accuracy  (%) | 98.1  [97.9, 98.1] | 98.0  [97.9, 98.2] | 98.8  [98.7, 98.8] | 98.7  [98.6, 98.8] | 99.5  [99.4, 99.5] | 99.5  [99.5, 99.6] | 99.5  [99.5, 99.5] | 99.4  [99.4, 99.5] | 99.6  [99.6, 99.6] | 99.5  [99.5, 99.6] |
| F-1 Score  (%) | 12.1  [11.4, 12.8] | 10.2  [8.7, 11.5] | 13.1  [11.9, 14.2] | 8.7  [7.0, 10.2] | 16.7  [14.8, 18.6] | 17.8  [13.8, 21.9] | 13.6  [12.0, 15.1] | 11.9  [8.4, 15.2] | 15.0  [13.0, 16.9] | 17.0  [13.5, 20.4] |
| AUPRC^b^  (%) | 26.5  [19.0, 34.1] | 14.2  [8.4, 22.7] | 34.7  [25.5, 44.0] | 14.4  [6.6, 26.0] | 45.0  [34.8, 55.0] | 46.5  [26.8, 66.5] | 50.5  [38.7, 61.6] | 35.4  [16.2, 56.9] | 35.4  [24.9, 46.6] | 18.4  [9.3, 30.6] |

^a^AUROC = area under receiver operating characteristic curve, ^b^AUPRC = area under precision-recall curve

Table G-1. This table presents the performance of the proposed model on patients with different age categories in training and testing subsets of the data. The numbers in the brackets are the estimated 95% confidence interval calculated by bootstrapping method.

### Appendix References

[1] Little, Roderick J., Ralph D'Agostino, Michael L. Cohen, Kay Dickersin, Scott S. Emerson, John T. Farrar, Constantine Frangakis et al. "The prevention and treatment of missing data in clinical trials." *New England Journal of Medicine* 367, no. 14 (2012): 1355-1360.
